# Longitudinal relationship between cognitive function and depressive symptoms in older adults: evidence from China

**DOI:** 10.3389/fpsyg.2026.1908800

**Published:** 2026-10-13

**Authors:** Hang Zhao, Xiao Mimi

**Affiliations:** School of Public Health, Chongqing Medical University, Chongqing, China

**Keywords:** cognitive function, depressive symptoms, fixed-effects model, longitudinal study, older adults

## Abstract

**Background:**

Longitudinal studies on cognitive function and depressive symptoms in Chinese older adults are relatively limited. This study aimed to investigate the effects of cognitive function on depressive symptoms in Chinese older adults.

**Methods:**

This study recruited 8,800 older adults from the China Health and Retirement Longitudinal Study (CHARLS) in 2011, 2013, 2015, 2018, and 2020. The Center for Epidemiologic Studies Depression Scale (CES-D10) was used to assess depressive symptoms, while mental status and situational memory were employed to evaluate cognitive function. Associations between cognitive function and current or subsequent depressive symptoms were analyzed using Pearson correlation analyses, and longitudinal relationships between cognitive function and depressive symptoms were analyzed using two-way fixed-effects models.

**Results:**

Pearson correlation analysis indicated a significant negative association between cognitive function and both current and subsequent depressive symptoms (*p* < 0.01). The two-way fixed-effects model results suggested that higher cognitive function was significantly associated with a lower risk of depressive symptoms after adjusting for all covariates (*β* = −0.124, 95% CI: −0.142, −0.106). Additionally, mental status had a stronger association on depressive symptoms compared to situational memory.

**Conclusion:**

In this study, higher cognitive function was significantly associated with lower depressive symptom scores in older adults. Relevant policymakers and stakeholders should develop cognitive function improvement programs to alleviate depressive symptom scores in older adults.

## Introduction

China, as the country with the largest elderly population, is experiencing the rapid process of aging. Aging has raised a series of public health problems, such as chronic diseases, cardiovascular diseases, mobility impairments, immune disorders, dementia and psychological disorders (Ćurko-Cofek, 2021; Zhou and Ma, 2022) burdening healthcare and elderly services. As one of the most common mental disorders among Chinese older adults, depressive symptoms have received widespread attention (Jiang et al., 2023). Depressive symptoms are mainly characterized by persistent sadness, loss of interest, and decreased ability to perform daily activities (Fried and Nesse, 2015). Based on a previous review, the prevalence of depressive symptoms in Chinese older adults was around 20% (Tang et al., 2021). Furthermore, depressive symptoms are associated with a range of negative experiences, including decreased quality of life, somatic symptoms, inflammation, suicide, sleep disorders, immune disorders, and economic burden (Mazza et al., 2022; Pandi-Perumal et al., 2020). Given the high prevalence and negative impact of depressive symptoms among Chinese older adults, it is necessary to identify potential influences on depressive symptoms.

Beyond mortality and morbidity, the concept of healthy and active aging has become central to public health discourse, emphasizing the preservation of physical function, cognitive capacity, emotional well-being, social participation, and quality of life in later life. Depressive symptoms represent a major threat to healthy aging, as they are associated with reduced functional autonomy, social withdrawal, and diminished quality of life, underscoring the importance of understanding their determinants across the life course.

With increasing age, older adults are inevitably at risk for impaired cognitive function, which typically manifests as impairment in one or more of the abilities related to perception, attention, memory, and computation (Taylor-Rowan et al., 2021). The relationship between cognitive function and depressive symptoms has been extensively investigated. Several studies have investigated the relationship between sub-dimensions of cognitive function and depressive symptoms and found that memory, executive function, and processing speed were significantly associated with depressive symptoms (Shimada et al., 2014). A cross-sectional survey from Chongqing, China, found that cognitive function and depressive symptoms were mutually influential factors (Giri et al., 2016). Furthermore, several studies have investigated mechanisms mediating the relationship between cognitive function and depressive symptoms, such as IADL disability, life satisfaction, and physical activity (Csajbok et al., 2022; Liu et al., 2023).

A previous study proposed the psycho-immune-neuroendocrine network model, which includes neuroinflammation, imbalances in the kynurenine pathway, hypothalamic–pituitary–adrenal axis hyperactivity, impaired neurotrophic support, and cerebrovascular dysfunction, contributing to an understanding of the relationship between cognitive function and depressive symptoms (Sapsford et al., 2022). Based on the stress theory, cognitive function impairment and related outcomes as potential stressors could potentially increase the risk of psychological distress and depression in older adults (Lu and Ruan, 2023). Meanwhile, impaired cognitive function is associated with decreased information processing and increased executive dysfunction (Jiang et al., 2023), impairing older adults’ ability to select, develop, and implement effective coping strategies (Gonda et al., 2015; Liu et al., 2015). Furthermore, impaired cognitive function may reduce social activities and increase the risk of social isolation in older adults (Gao et al., 2022), which would increase the risk of depressive symptoms.

Beyond biological pathways, a conceptual sequence may be proposed: cognitive impairment leads to difficulties in coping with problems and maintaining activities of daily living, which in turn reduces social participation and physical activity, increases social isolation and lowers life satisfaction, and ultimately elevates the risk of depressive symptoms. This pathway highlights the importance of examining cognitive function as a modifiable upstream determinant of depressive symptoms in older adults.

Although previous studies have investigated the cross-sectional relationship between cognitive function and depressive symptoms, most have focused on the impact of depressive symptoms on cognitive function (Gao et al., 2022). Limited academic exploration has addressed the longitudinal association between cognitive function and depressive symptom scores in older adults (Lu and Ruan, 2023), especially in the context of China’s rapid aging. Simultaneously, most studies examining the relationship between cognitive function and depressive symptoms have relied on localized samples or cross-sectional surveys, possibly limiting conclusion generalization and longitudinal relationships. Therefore, this study examined the longitudinal relationship between cognitive function and depressive symptom scores in older adults using nationally representative longitudinal data, providing an empirical basis for improving mental health in older adults.

## Methods

### Data sources and study population

The data used in this study were derived from the China Health and Retirement Longitudinal Survey (CHARLS), which was designed to collect microdata on middle-aged and older adults in China, including sociodemographic information, household information, health status, health behaviors, insurance coverage, etc. The CHARLS project conducted the first national baseline survey in 2011, and follow-up surveys were conducted in 2013, 2015, 2018, and 2020. The CHARLS program covered 28 provinces across the country using probability proportional to size sampling. The investigation was approved by the Ethics Review Board of Peking University (IRB00001052-11015), and all participants signed the written informed consent. A detailed description of CHARLS has been published elsewhere (Zhao et al., 2014).

The five waves of CHARLS data were selected for this study to investigate the relationship between cognitive function and depressive symptom scores in older adults. Study participants were screened according to the following exclusion criteria: (1) age younger than 60; (2) missing information on cognitive function or depressive symptoms; (3) participants with only one survey; and (4) cognitive and depression-related illnesses such as stroke, cerebral infarction, dementia, and Parkinson at the first survey. Finally, this study yielded unbalanced panel data for 8,800 individuals with 27,402 samples, including 4,460 participants in 2011, 5,121 participants in 2013, 6,702 participants in 2015, 5,360 participants in 2018, and 5,759 participants in 2020.

### Measurement

The 10-item Center for Epidemiologic Studies Depression Scale (CES-D10) was used to measure depressive symptom scores in older adults. Older adults were asked 10 questions about feelings and behaviors in the past week, with four options for each question: 0 = never, 1 = rarely or sometimes, 2 = sometimes, and 3 = always. The questions “hopeful for the future” and “very happy” were reverse scored (Cai et al., 2024). The total CES-D10 score ranged from 0 to 30, with higher scores indicating more severe depressive symptoms. The CES-D10 has been widely used to measure depressive symptoms in Chinese older adults with good reliability and validity (Zheng et al., 2023). Referring to previous studies, older adults with CES-D10 scores greater than 10 were defined as having depressive symptoms (Feng et al., 2021).

The CHARLS program used mental status and situational memory to measure cognitive function (Pan et al., 2022). Mental status included the Telephone Interview of Cognitive Status (TICS) and the drawing test, with values ranging from 0 to 11. TICS assessed time orientation by investigating the current month, day, year, week, and season, and numerical ability by subtracting 7 continuously from 100 (up to 5 times). The drawing test evaluated visuospatial ability by asking respondents to imitate drawing two overlapped pentagons. Situational memory was measured by immediate and delayed memory, with values ranging from 0 to 20. Respondents were asked to immediately repeat the 10 Chinese nouns they had just heard and to recall the same list of words 4 min later (Zhao et al., 2023). Cognitive function was summed from mental state and situational memory and ranged from 0 to 31, with higher scores indicating better cognitive function (Ding et al., 2023).

Covariables were included in this study for controlling potential confounders. Sociodemographic characteristics included age, gender (male and female), hukou status (urban and rural), marital status (married and other), and education level (illiterate, primary and below, and middle school and above). Health status included self-reported health (poor, fair, and good), chronic disease (yes and no), pain (yes and no), basic activities of daily living (BADL), and instrumental activities of daily living (IADL). Respondents were considered to have BADL impairment if they had difficulty dressing, bathing, eating, getting in and out of bed, going to the toilet, and controlling bowel movements. Respondents were considered to have IADL impairment if they had difficulty with housework, cooking, shopping, taking medication, and managing money. Health behaviors included sleep duration (7–9 h/day and <7 or >9 h/day) (Yao et al., 2022), social activities (yes and no), smoking (yes and no), and drinking (yes and no). Social security factors included health insurance (yes and no) and pensions (yes and no).

### Statistical analysis

All statistical analyses were performed using STATA 17.0 and IBM SPSS Statistics 26.0. Covariates with missing values were filled in using multiple interpolations to minimize selection bias and improve statistical efficacy (Cai et al., 2024; Sun et al., 2022). Frequencies and percentages were used to describe categorical variables, while means and standard deviations were used to describe continuous variables. Associations between cognitive function and depressive symptoms across time were tested using Pearson correlation analysis. Two-way fixed-effects models were employed to estimate the relationship between cognitive function and depressive symptom scores in older adults. Two-way fixed-effects models improved estimation accuracy by controlling for individual characteristics that did not vary over time and time characteristics that varied over time. Five models were used in this study to demonstrate the longitudinal relationship between cognitive function and depressive symptoms. Model 1 was considered unadjusted. Model 2 was adjusted for sociodemographic characteristics. Model 3 was adjusted for sociodemographic characteristics and health status. Model 4 was adjusted for sociodemographic characteristics, health status, and health behaviors. Model 5 was adjusted for sociodemographic characteristics, health status, health behaviors, and social security factors. Furthermore, this study analyzed the relationship between sub-dimensions of cognitive function and depressive symptoms. All models employed robust standard errors to avoid potential heteroskedasticity problems. The variance inflation factor (VIF) was used to determine the co-linearity problem.

## Results

### Participants’ characteristics

Table 1 shows the basic characteristics of the participants. The average age of the participants increased from 66.75 in 2011 to 70.10 in 2020. In the five waves of the survey, the majority of participants were male (52.69–56.42%), were from rural areas (69.70–74.19%), were married (81.19–83.88%), had chronic diseases (72.30–86.80%), had an unimpaired BADL (74.72–78.54%), had an unimpaired IADL (76.26–80.59%), slept too many or too few hours (57.62–66.24%), did not drink alcohol (63.12–65.40%), and had health insurance (88.11–97.84%). Approximately half had an education level of primary school or below (47.69–51.20%). The proportion of participants with good self-reported health and pensions increased between 2011 and 2020. The ratio of participants’ social engagement and smoking status remained roughly 1:1.

**Table 1:** Characteristics of participants in 2011, 2013, 2015, 2018 and 2020.

Variables	2011 (*N* = 4,460)	2013 (*N* = 5,121)	2015 (*N* = 6,702)	2018 (*N* = 5,360)	2020 (*N* = 5,759)
Age	66.75 (5.69)	67.09 (5.86)	67.73 (6.08)	68.17 (5.81)	70.10 (5.66)
Gender					
Male	2,383 (53.43)	2,869 (56.02)	3,531 (52.69)	3,024 (56.42)	3,089 (53.64)
Female	2,077 (46.57)	2,252 (43.98)	3,171 (47.31)	2,336 (43.58)	2,670 (46.36)
Hukou status					
Urban	1,151 (25.81)	1,178 (28.86)	1,766 (26.35)	1,613 (30.09)	1,745 (30.30)
Rural	3,309 (74.19)	3,643 (71.14)	4,936 (73.65)	3,747 (69.91)	4,014 (69.70)
Marriage					
Married	3,687 (82.67)	4,284 (83.66)	5,496 (82.01)	4,496 (83.88)	4,676 (81.19)
Other	773 (17.33)	837 (16.34)	1,206 (17.09)	864 (16.12)	1,083 (18.81)
Education					
Illiteracy	1,300 (29.15)	1,192 (23.28)	1,817 (27.11)	840 (15.67)	1,154 (20.04)
Primary and below	2,154 (48.30)	2,622 (51.20)	3,196 (47.69)	2,660 (49.63)	2,763 (47.98)
Middle school and above	1,006 (22.56)	1,307 (25.52)	1,689 (25.20)	1,860 (34.70)	1,842 (31.98)
Self-reported health					
Poor	2,367 (53.07)	2,571 (50.21)	3,511 (52.39)	1,345 (25.09)	1,466 (25.46)
Fair	1,456 (32.65)	1,739 (33.96)	2,105 (31.41)	2,775 (51.77)	3,035 (52.70)
Good	637 (14.28)	811 (15.84)	1,086 (16.20)	1,240 (23.13)	1,258 (21.84)
Chronic					
Yes	3,269 (72.30)	4,124 (80.53)	5,610 (83.71)	4,597 (85.76)	4,999 (86.80)
No	1,191 (27.70)	997 (19.47)	1,092 (16.29)	763 (14.24)	760 (13.20)
BADL					
Impaired	968 (21.70)	1,099 (21.46)	1,694 (25.28)	1,177 (21.96)	1,370 (23.79)
Unimpaired	3,492 (78.30)	4,022 (78.54)	5,008 (74.72)	4,183 (78.04)	4,389 (76.21)
IADL					
Impaired	942 (21.12)	994 (19.41)	1,591 (26.74)	1,128 (21.04)	1,289 (22.38)
Unimpaired	3,518 (78.88)	4,127 (80.59)	5,111 (76.26)	4,232 (78.96)	4,470 (77.62)
Pain					
Yes	1,406 (31.52)	1,738 (33.94)	1,985 (29.62)	3,193 (59.57)	3,361 (58.36)
No	3,054 (68.48)	3,383 (66.06)	4,717 (70.38)	2,167 (40.43)	2,398 (41.64)
Sleep duration					
7–9 h/day	1,890 (42.38)	1,988 (38.82)	2,732 (40.76)	2,066 (38.54)	1,944 (33.76)
<7 or >9 h/day	2,570 (57.62)	3,133 (61.18)	3,970 (59.24)	3,294 (61.46)	3,815 (66.24)
Social activity					
Yes	2,296 (51.48)	3,015 (58.88)	3,454 (51.54)	2,947 (54.98)	2,794 (48.52)
No	2,164 (48.52)	2,106 (41.12)	3,248 (48.46)	2,413 (45.02)	2,965 (51.48)
Smoking					
Yes	1,965 (44.06)	2,594 (50.65)	3,062 (45.69)	2,633 (49.12)	2,542 (44.14)
No	2,495 (55.94)	2,527 (49.35)	3,640 (54.31)	2,727 (50.88)	3,217 (55.86)
Drinking					
Yes	1,448 (32.47)	1,851 (36.15)	2,319 (34.60)	1,977 (36.88)	2,109 (36.62)
No	3,012 (67.53)	3,270 (63.85)	4,383 (65.40)	3,383 (63.12)	3,650 (63.38)
Medical insurance					
Yes	4,225 (94.73)	4,975 (97.15)	5,905 (88.11)	5,244 (97.84)	5,529 (96.01)
No	235 (5.27)	146 (2.85)	797 (11.89)	116 (2.16)	673 (3.99)
Pension					
Yes	2,694 (39.60)	4,246 (82.91)	5,023 (74.95)	4,981 (92.93)	5,086 (88.31)
No	1,766 (60.40)	875 (17.09)	1,679 (25.05)	379 (7.07)	673 (11.69)

### Pearson’s analysis of the association between cognitive function and depressive symptoms over time

Table 2 reports the Pearson correlation analysis between cognitive function and depressive symptoms. The results showed that there was a significant correlation between current cognitive function and both current and subsequent depressive symptoms (*p* < 0.01). Specifically, the correlation coefficient (r) ranged from −0.296 to −0.237 for current cognitive function and current depressive symptoms, and from −0.233 to −0.190 for current cognitive function and subsequent depressive symptoms. Therefore, cognitive function was significantly associated with both current and subsequent depressive symptoms without adjusting for potential confounders.

**Table 2:** Results of Pearson analysis of cognitive function and depressive symptoms across different years.

Variables	Dep2011	Cog2011	Dep2013	Cog2013	Dep2015	Cog2015	Dep2018	Cog2018	Dep2020	Cog2020
Dep2011	1									
Cog2011	−0.296^***^	1								
Dep2013	0.504^***^	−0.193^***^	1							
Cog2013	−0.227^***^	0.564^***^	−0.237^***^	1						
Dep2015	0.492^***^	−0.242^***^	0.547^***^	−0.208^***^	1					
Cog2015	−0.217^***^	0.607^***^	−0.190^***^	0.596^***^	−0.274^***^	1				
Dep2018	0.471^***^	−0.224^***^	0.504^***^	−0.216^***^	0.550^***^	−0.235^***^	1			
Cog2018	−0.224^***^	0.553^***^	−0.206^***^	0.534^***^	−0.226^***^	0.621^***^	−0.278^***^	1		
Dep2020	0.474^***^	−0.243^***^	0.480^***^	−0.230^***^	0.533^***^	−0.264^***^	0.568^***^	−0.257^***^	1	
Cog2020	−0.179^***^	0.555^***^	−0.200^***^	0.519^***^	−0.225^***^	0.624^***^	−0.233^***^	0.617^***^	−0.290^***^	1

*p < 0.1, **p < 0.05, ^***^p < 0.01; Dep., depression; Cog., cognition.

### Longitudinal relationship between cognitive function and depressive symptoms

Table 3 presents the results of the stepwise two-way fixed effects model. The VIF for all models was less than 2, suggesting the absence of the co-linearity problem (Sun et al., 2021). The estimated coefficients of cognitive function and depressive symptom scores in older adults in Models 1, 2, 3, 4, and 5 were −0.139 (95% CI: −0.157, −0.120), −0.136 (95% CI: −0.155, −0.118), −0.126 (95% CI: −0.144, −0.108), −0.125 (95% CI: −0.143, −0.106), and −0.124 (95% CI: −0.142, −0.106). These results suggested that higher cognitive function was significantly associated with a lower risk of depressive symptoms after controlling for potential confounders. With the increase in covariates, the R-squared of the two-way fixed effects model increased from 0.027 to 0.074, indicating improved explanatory power. Additionally, urban hukou, being married, good self-rated health, normal sleep duration, participation in social activities, and having pensions were significantly associated with a lower risk of depressive symptoms, while chronic illness, impaired BADL, and impaired IADL were significantly associated with a higher risk of depressive symptoms (*p* < 0.05). Although individual fixed effects generally absorb time-invariant characteristics, we report the coefficient for gender as estimated from the between-individual variation in model specification. Readers should note that the interpretation of time-invariant covariates in fixed-effects models differs from standard regression coefficients, and these estimates should be interpreted with appropriate caution.

**Table 3:** Two-way fixed-effects model analysis of cognitive function and depressive symptoms.

Variables	Depression				
	Model 1	Model 2	Model 3	Model 4	Model 5
Cognitive function	−0.139^***^	−0.136^***^	−0.126^***^	−0.125^***^	−0.124^***^
	(−0.157, −0.120)	(−0.155, −0.118)	(−0.144, −0.108)	(−0.143, −0.106)	(−0.142, −0.106)
Age		−0.014	−0.016	−0.020	−0.020
		(−0.086, 0.059)	(−0.085, 0.053)	(−0.089, 0.049)	(−0.088, 0.049)
Gender (ref: male)					
Female		−1.625^**^	−1.536^**^	−1.560^**^	−1.553^**^
		(−3.140, −0.110)	(−3.008, −0.064)	(−3.033, −0.088)	(−3.023, −0.083)
Hukou status (ref: urban)					
Rural		−0.883^***^	−0.871^***^	−0.866^***^	−0.864^***^
		(−1.294, −0.473)	(−1.274, −0.468)	(−1.268, −0.464)	(−1.266, −0.462)
Marriage (ref: married)					
Other		−1.263^***^	−1.177^***^	−1.166^***^	−1.161^***^
		(−1.690, −0.837)	(−1.591, −0.764)	(−1.579, −0.753)	(−1.573, −0.748)
Education (ref: illiteracy)					
Primary and below		0.151	0.150	0.161	0.156
		(−0.307, 0.609)	(−0.293, 0.593)	(−0.282, 0.603)	(−0.286, 0.598)
Middle school and above		0.100	0.137	0.149	0.143
		(−0.494, 0.693)	(−0.439, 0.713)	(−0.427, 0.725)	(−0.433, 0.719)
Self-reported health (ref: poor)					
Fair			−0.681^***^	−0.667^***^	−0.667^***^
			(−0.836, −0.525)	(−0.823, −0.512)	(−0.822, −0.512)
Good			−1.336^***^	−1.326^***^	−1.326^***^
			(−1.537, −1.135)	(−1.527, −1.124)	(−1.528, −1.125)
Chronic (ref: no)					
Yes			0.349^***^	0.347^***^	0.351^***^
			(0.099, 0.599)	(0.097, 0.597)	(0.101, 0.602)
BADL (ref: impaired)					
Unimpaired			0.959^***^	0.947^***^	0.945^***^
			(0.773, 1.145)	(0.762, 1.133)	(0.759, 1.131)
IADL (ref: impaired)					
Unimpaired			1.029^***^	1.022^***^	1.020^***^
			(0.828, 1.229)	(0.821, 1.222)	(0.820, 1.221)
Pain (ref: no)					
Yes			1.012^***^	1.000^***^	1.002^***^
			(0.854, 1.170)	(0.842, 1.159)	(0.844, 1.161)
Sleep duration (ref: <7 or >9 h/day)					
7–9 h/day				−0.527^***^	−0.528^***^
				(−0.675, −0.378)	(−0.676, −0.380)
Social activity (ref: no)					
Yes				−0.151^**^	−0.148^**^
				(−0.295, −0.007)	(−0.292, −0.004)
Smoking (ref: no)					
Yes				−0.062	−0.056
				(−0.433, 0.308)	(−0.426, 0.314)
Drinking (ref: no)					
Yes				−0.115	−0.109
				(−0.321, 0.090)	(−0.315, 0.096)
Medical insurance (ref: no)					
Yes					0.026
					(−0.288, 0.340)
Pension (ref: no)					
Yes					−0.200^**^
					(−0.368, −0.033)
Constant	10.104^***^	13.085^***^	12.477^***^	13.103^***^	13.112^***^
	(9.797, 10.412)	(8.381, 17.789)	(7.987, 16.967)	(8.612, 17.593)	(8.608, 17.616)
Individual fixed effect	Yes	Yes	Yes	Yes	Yes
Year fixed effects	Yes	Yes	Yes	Yes	Yes
Observation	27,402	27,402	27,402	27,402	27,402
R-square	0.027	0.031	0.071	0.074	0.074

*p < 0.1, **p < 0.05, ^***^p < 0.01; BADL, basic activities of daily living; IADL, instrumental activities of daily living.

### Relationship between sub-dimensions of cognitive function and depressive symptoms

Table 4 presents the relationship between cognitive function and its sub-dimensions with depressive symptoms. The three models were adjusted for all control variables. The estimated coefficients for cognitive function, mental status, situational memory, and depressive symptoms were −0.124 (95% CI: −0.142, −0.106), −0.158 (95% CI: −0.191, −0.125), and −0.123 (95% CI: −0.146, −0.101), respectively. These results indicated that sub-dimensions of cognitive function also have significant effects on depressive symptoms. Relative to situational memory, mental state had a stronger association on depressive symptom scores in older adults.

**Table 4:** Two-way fixed-effects analysis of cognitive function and its sub-dimensions with depressive symptoms.

Variables	Depression		
Cognitive function	−0.124^***^		
	(−0.142, −0.106)		
Mental status		−0.158^***^	
		(−0.191, −0.125)	
Situational memory			−0.123^***^
			(−0.146, −0.101)
Covariates	Yes	Yes	Yes
Individual fixed effect	Yes	Yes	Yes
Year fixed effects	Yes	Yes	Yes
Constant	13.112^***^	12.642^***^	12.030^***^
Observation	(8.608, 17.616)	(8.110, 17.174)	(7.544, 16.516)
R-square	0.074	0.069	0.071

*p < 0.1, **p < 0.05, ^***^ *p* < 0.01; The three models were adjusted for all covariates.

## Discussion

This study examined the longitudinal association between cognitive function and depressive symptom scores in older adults using two-way fixed-effects models and CHARLS 2011–2020 unbalanced panel data. The results showed that higher cognitive function, mental status, and situational memory in older adults were significantly associated with a lower risk of depressive symptoms after controlling for confounders. Compared to situational memory, mental status had a stronger association on depressive symptom scores in older adults. To our knowledge, this study is one of the few longitudinal investigations examining the longitudinal association between cognitive function and depressive symptoms in Chinese older adults, contributing to filling gaps in existing research and informing public health policy.

This study found that cognitive function was significantly and negatively associated with depressive symptom scores in older adults, consistent with previous research (Jiang et al., 2023; Zhang et al., 2020). Although the relationship between cognitive function and depressive symptoms has been investigated by several scholars, no consistent conclusions have been reached. A cross-sectional survey from Korea found that cognitive function was an important influence on depressive symptom scores in older adults (Yun and Bae, 2020), while a longitudinal survey concluded that cognitive decline was also an influence on depressive symptom scores in older adults (Yang et al., 2023). Furthermore, several studies have emphasized the impact of a certain domain of cognitive function on depressive symptom scores in older adults, such as immediate memory, delayed memory, executive function, and attention (Knight et al., 2020). However, a few studies have shown that cognitive function does not predict depressive symptom scores in older adults. For instance, a longitudinal survey from Canberra found that cognitive function failed to predict changes in depressive symptom scores over time (Bunce et al., 2014). These different results may be due to differences in study design, survey populations, measurement tools, analytic methods, and cultural contexts, and more causal inferences can be performed in the future.

It is also important to acknowledge the possibility of a bidirectional relationship between cognitive function and depressive symptoms. While this study examined the association of cognitive function with subsequent depressive symptom scores, depressive symptoms may in turn adversely affect cognitive performance. Future studies using cross-lagged panel models or instrumental variable approaches would be better positioned to disentangle the directionality of this relationship. The findings of the present study should therefore be interpreted as reflecting longitudinal within-person associations rather than unidirectional causal effects.

There are several potential pathways through which cognitive function is longitudinally associated with depressive symptom scores in older adults. At the biological level, the association between cognitive function and depressive symptom scores in older adults probably involved several common biological mechanisms, including vascular disease, altered levels of glucocorticoid steroids and hippocampal atrophy, increased deposition of amyloid-β plaques, inflammatory changes, and nerve growth factor deficiency (Zhou et al., 2021). At the psychological level, cognitive decline may be associated with result in impairments in information processing, memory, attention, and executive functions in older adults, which will affect their self-efficacy and sense of self-worth (Klůzová Kráčmarová et al., 2020), thereby increasing the risk of depression (Rippon et al., 2022). At the social level, impaired cognitive function may hinder communication and social activities for older people, reducing their social participation (Donkers et al., 2019). Meanwhile, cognitive decline may be associated with lead to changes in social roles or status, resulting in social exclusion or discrimination and increasing psychological stress (Hossain et al., 2022). Therefore, the relationship between cognitive function and depressive symptom scores in older adults is complex and multifactorial. Future research could further explore the potential pathways of this relationship, including biological, psychological, and social dimensions.

The present study found that mental status showed a stronger longitudinal association with depressive symptom scores in older adults compared to situational memory. There are several possible explanations. Firstly, mental state encompasses orientation, computation, attention, and visuospatial abilities, providing more information than situational memory, which may account for differences in estimated coefficients. Secondly, the decline in mental status has more pronounced effects on individuals and their families, leading to greater psychological distress compared to situational memory. Finally, the sensitivity of the measurement instrument may have contributed to differences in the estimated coefficients, as mental status is more likely to capture early changes in cognitive function. The finding could be validated in the future using more statistical models.

This study enhances the understanding of the longitudinal association between cognitive function and depressive symptoms in Chinese older adults, providing empirical experience for the development of depressive symptom intervention policies. However, this study has some limitations. Firstly, the measures of cognitive function and depressive symptoms in this study were based on validated screening instruments rather than on clinical diagnostic criteria. Future studies could investigate the relationship between cognitive function and depressive symptom scores in older adults based on clinical criteria. Secondly, although this study controlled for many confounding factors, there were still some omitted variables that could have influenced the results, such as biological factors, genetic factors, and personality traits. Finally, the information related to this study was based on self-reported assessments by the respondents, which may suffer from recall bias and lead to biased estimates.

## Conclusion

This study examined the longitudinal association between cognitive function and depressive symptom scores in older adults using two-way fixed-effects models and nationally representative unbalanced panel data, addressing the limitations of previous studies that relied on cross-sectional or localized surveys. Notably, older adults with impaired cognitive function, mental status, and situational memory were at increased risk for depressive symptoms. Therefore, monitoring and improving cognitive function in older adults is crucial for identifying potential episodes of depressive symptoms. The study emphasizes the importance of developing and implementing comprehensive prevention and treatment strategies, such as cognitive stimulation, cognitive training, and cognitive rehabilitation (Bahar-Fuchs et al., 2013), to mitigate the impact of future psychological stress and disease burden.

## Data Availability

The original contributions presented in the study are included in the article/supplementary material, further inquiries can be directed to the corresponding author.
